# Bio-event definition in text mining towards event interconnection

**DOI:** 10.1186/1753-6561-9-S5-A5

**Published:** 2015-08-06

**Authors:** Chen Li, Maria Liakata

**Affiliations:** 1 Computer Science and Artificial Intelligence Laboratory, Massachusetts Institute of Technology, MA, US; 2 Department of Computer Science, University of Warwick, Coventry, UK

## Introduction

Event extraction is one of the main focuses in bio-text mining (TM). Interconnecting extracted events into reaction networks provides biologists with a wealth of fine-grained information on biochemical reactions [1]. Intuitively, extracted events could be connected into networks based on common entities. However, this approach is limited due to: 1) its dependence on flawless entity normalisation; 2) inability to express the directionality of the various relations/reactions. To enrich the information in extracted events and facilitate their interconnection, we propose a modification to bio-event definition to make it more compatible with the structure of biological reactions and community-supported biological semantic resources. More specifically, we propose alignment of bio-events with the reactions in the Systems Biology Markup Language (SBML), which would make bio-events more biologically meaningful and directly re-usable by domain experts.

## Background

The last decade has seen increased interest and rapid advance in the semantic study of biology, resulting in a number of semantic knowledge resources proposed by the bio community. The Systems Biology Markup Language (SBML) [2] is a successful example of such efforts. SBML is a machine readable and transferable format depicting biological/biochemical reaction networks. It has been widely adopted and has been used for encoding a broad range of biological networks.

Ohta et al. [3] compared SBML with the event definitions in the series of GENIA tasks, pointing out that bio-event types in the current TM tasks are insufficient for covering all types of biochemical reactions in existing networks. The latter tasks in the BioNLP series try to expand the coverage of more types of bio-events [4].

We argue below that not only would we require more types of events but also a modification to current event structure.

## BioNLP events and biological reactions

The main arguments of an event defined in the latest BioNLP'13 GE task consist of *Theme *and *Cause *[5]. While *Theme *and *Cause *have a direct correspondence to the notions of *Patient *and *Agent *respectively, in linguistics thematic relations, assignment of these arguments delivers insufficient information about the roles of participants in reactions.

**Figure 1:**
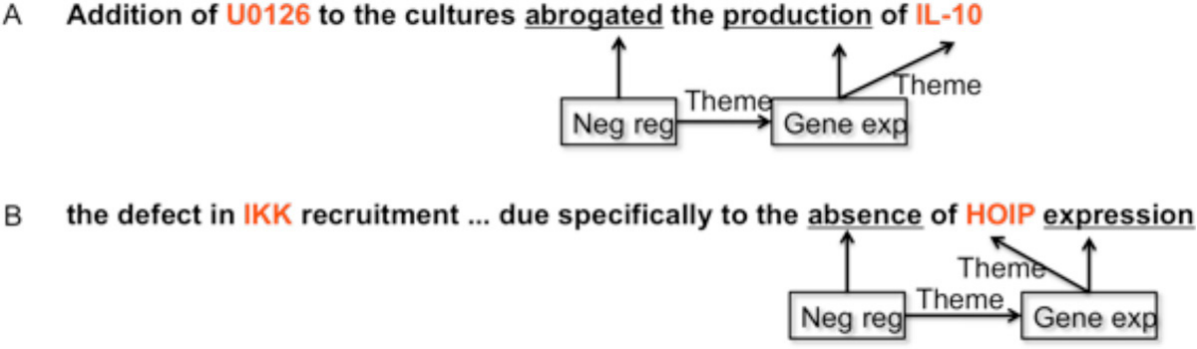
**An example of *Theme *and *cause *illustrates that the directionality of event is missing**.

Consider the following two sentences annotated with BioNLP events, shown in Figure 1. The *gene expression*, GE1, in sentence A is a *theme *of a *negative regulation*. In sentence B, the *gene expression*, GE2, also is a *theme *of a *negative regulation*. However, the roles of the two gene expression (GE) events are very different, with GE1 being passively regulated in contrast to GE2, which is actually regulating another event in the sentence.

By contrast, when we look at an SBML file, it encodes a network as a set of biochemical reactions interconnected by the participants. The main elements of each reaction include reactants, modifiers and products which respectively denote the substances involved in, influencing and produced by the reaction.

The BioNLP 2013 Pathway Curation (PC) task [6] has augmented the Theme and Cause arguments by including Products and Participants. However, several issues remain unresolved. For example, a protein modification event in the PC task contains a single *Theme*. This is based on the knowledge that such events occur between proteins and certain molecules, which always result in the binding of the two. Without explicitly mentioning the products though, computers would not be able to automatically interconnect such events via, for example, coreference. Moreover, regulatory events cannot be incorporated into a gene expression event as modifiers if the event is not provided in a directional format. Modifiers are also missing for many events.

**Figure 2:**
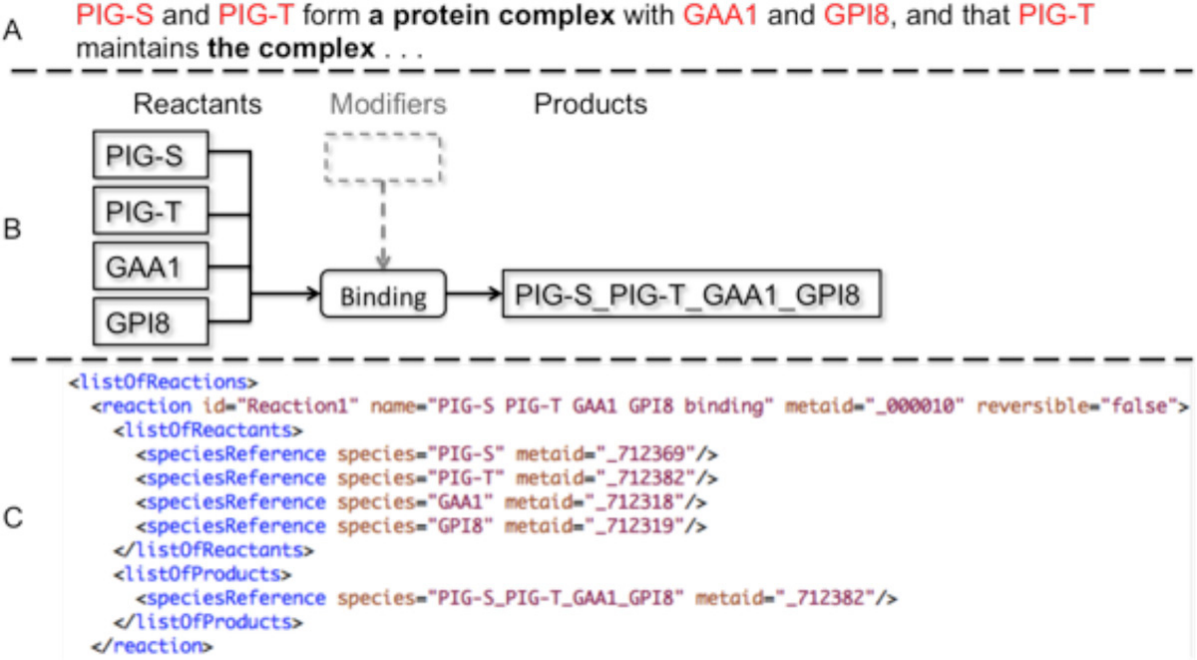
**An example of SBML**. **A **is the sentence describing the process. **B **is the diagram depicting the three main elements of an SBML reaction, which are reactants, modifiers and products. No modifier is involved in the example reaction (grey dash line). **C **is the SBML snippet encoding the binding process.

Figure 2 shows how an SBML reaction is abstracted from text. The example uses an inferred protein complex to facilitate future sensible entity coreference, as a protein complex in the first clause refers to the complex in the second clause. Therefore, if we tune event extraction output to distinguish between reactants, products and modifiers, the output will be more biologically informative and enable easier event interconnection.

## Proposed amendments to event definition

We use examples selected from the BioNLP'13 GE task and illustrate a possible extension to the current format for all event types in the task (Table 1). This can be extended to other tasks. The format focuses on enhancing the information capabilities of *theme *and *cause*. It could be potentially used for mapping the existing BioNLP annotations to the more SBML-compatible format.

**Table 1:** Examples for the event types from BioNLP'13 GE task.

	Reactants	Modifiers	Products	Example
Gene exp	IL-10		IL-10	IL-10 production

Transcription	MBP mRNA			MBP mRNA transcription

Protein cata	p100			degradation of the p100 NF-kB protein

Binding	HOIP, CD40		HOIP CD40 (inferred)	the association of HOIP with CD40

Localization	ΔFKH			localization of ΔFKH

Protein modi	p65			Post-translational modification of NF-kB p65

Phosphorylat	NF-kB		NF-kB pho (inferred)	NF-kB p65 phosphorylation

Ubiquitinati	I-kB*α*		I-kB*α *ubi (inferred)	ubiquitination, and subsequent degradation of I-kB*α*

Acetylation	p65		p65 ace (inferred)	Acetylation of p65

Deacetylation	p65	histone deacetylase-3	p65 dea (inferred)	Deacetylation of p65 by histone deacetylase-3

Regulation	Ser536 TRAF2	HOIP		Example 1: point mutation at Ser536 Example 2: HOIP functions downstream of TRAF2

Positive reg	PKC*α*	M-CSF		M-CSF stimulated PKC*α*

Negative reg	MEK1, MEK2			inhibits MEK1 and MEK2

*Gene expression *(GE) is the process of synthesizing proteins from genetic codes. The same term is used to refer to a gene and gene product, e.g. protein. Therefore, the same gene name is used for both reactant and product. As a sub-process of GE, *Transcription *could take the same approach. So, the GE example in Table 1 could have IL-10 as the reactant.

*Protein catabolism *is the process of proteins breaking into amino acids. Therefore, if the mentions of generated amino acids appear along a broken protein, the amino acid names should be annotated as products. Meanwhile, if the names of related proteases occur, they should be annotated as modifiers.

*Binding *is the formation of macromolecules by the aggregation of two or more molecules. Generated molecules, physical clusters of the original macro-molecules will constitute the products. In Table 1, the inferred cluster could be named after the conjunction of the reactants. This can help event interconnections to produce more sensible reaction cascades. For example, in "post-translational modification state of CD40-associated HOIP", post-translational modification is taking place on the macromolecule consisting of CD40 and HOIP instead of either of them. If the product of the *binding *of CD40 and HOIP is named as CD40 HOIP, the downstream encoding of *protein modification *can use CD40 HOIP as the reactant.

The more specific *Protein modification *types include *phosphorylation, ubiquitination, acetylation*, and *deacetylation*. These processes attach specific chemicals onto other molecules. Therefore, the products of these processes can be inferred in a similar way as for binding.

*Regulations *including *positive *and *negative regulations *are the processes, which catalyze or inhibit other processes without producing anything via the actual regulation process per se. They are akin to the notion of modifiers defined in SBML. We propose that regulations should be incorporated into the processes they have influenced. This would merge *regulation *events with others. For example, "Addition of U0126 to the cultures abrogated the production of IL-10" could be extracted as a *gene expression *event of IL-10 with U0126 as the modifier rather than an extra *regulation *event of U0126, although the extraction may be technically achieved in two steps.

## Discussion

Event interconnection requires further research into entity coreference, event coreference and discourse analysis. Encoding extracted and inferred information from bio-events into SBML format can help by maintaining reaction directionality and enabling meaningful coreference.

This position paper argues that it is possible and indeed advantageous to enhance the output formats of extracted bio-events and make them compatible with the widely used SBML format for biological reactions. The format can be further refined to meet the complexity of bio-events. A possible first step would be to use the enhanced format to annotate existing corpora, e.g. those from BioNLP tasks or adapt them to the new format semi-automatically.
